# *CALR* Mutation Underlying Silent Stroke

**DOI:** 10.1055/s-0041-1728674

**Published:** 2021-06-01

**Authors:** Rehman Faryal, Lisa Lee Tokar, Stephen E. Langabeer, Janusz Krawczyk

**Affiliations:** 1 Department of Haematology, Galway University Hospital, Galway, Ireland; 2 Cancer Molecular Diagnostics, St. James's Hospital, Dublin, Ireland

Essential thrombocythemia (ET) is one of the classical Philadelphia chromosome-negative myeloproliferative neoplasms (MPN) characterized by a high platelet count and a tendency for thrombotic and/or hemorrhagic events. 1 ET is driven by acquired mutations in either the *JAK2* , *CALR* , or *MPL* genes with the *JAK2* V617F mutation a significant risk factor for development of stroke in patients with MPN. 2 In contrast, *CALR* -mutated ET is associated with higher platelet counts but a lower risk of thrombotic events than their *JAK2* V617F-positive counterparts. 3 Furthermore, patients with type 1 *CALR* mutations (52 base-pair deletion) appear to have an increased risk of thrombosis compared with patients harboring type 2 *CALR* mutations (five base-pair insertion). 4 5 This aspect has been further illustrated in the molecular screening of large cohorts of patients with stroke (with and without an hematologically overt MPN) that have shown no evidence of the common ET-associated *CALR* mutation types. 6 7

**Fig. 1:**
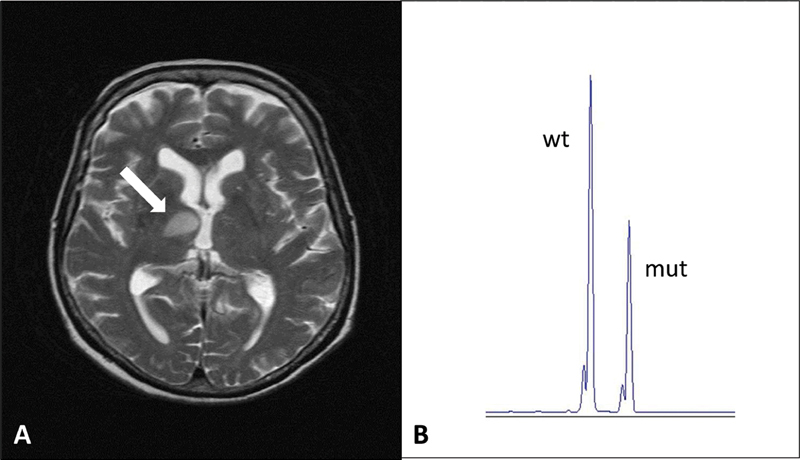
( **A** ) Brain magnetic resonance imaging depicting acute infarct (arrow). ( **B** ) Capillary electrophoresis after fragment length analysis showing a wild-type 263 base-pair peak and mutant 268 base-pair peak in *CALR* exon 9.

A 78-year-old female presented in the emergency department with a new onset parieto-occipital headache and recurring episodes of dizziness. She was recently diagnosed with hypertension, which was well controlled with an antihypertensive. On examination, she was vitally stable with no neurological findings. Computed tomography (CT) scan of the brain showed an area of hypoattenuation in the right thalamus that was confirmed as an acute infarct by magnetic resonance imaging (MRI) ( Fig. 1A ). Cardiac workup including carotid Doppler that revealed patent carotid arteries and echocardiography that demonstrated good systolic function, no valvular defects and normal atrial dimensions. The patient remained on telemetry for rhythm monitoring as part of the stroke workup that suggested normal sinus rhythm. In view of the CT and MRI findings and lack of any obvious neurological deficit, the picture was consistent with a “silent” stroke. Hematological workup showed hemoglobin of 13.3 g/dL, white cell count of 7.5 × 10 ^9^ /L and platelet count of 1,163 × 10 ^9^ /L (normal range = 150–400 × 10 ^9^ /L). The blood film showed platelet anisocytosis, but no evidence of dacrocytes. A thrombophilia screen was not indicated according to local and national guidelines given the patients' age, no history of thrombosis and no family history of thrombosis. The patient refused a bone marrow biopsy with the diagnosis more consistent with ET rather than pre-fibrotic myelofibrosis due to the absence of anemia, leukocytosis, a leucoerythroblastic blood film, palpable splenomegaly, and a raised lactate dehydrogenase (200 IU/L; normal range 135–214 IU/L). 8 The *JAK2* V617F mutation was not detected, but fragment length analysis followed by capillary electrophoresis revealed the presence of the common five base-pair insertion (type 2) mutation in *CALR* exon 9 at an allele frequency of 36.2%, providing further evidence of ET ( Fig. 1B ). After consultation with stroke team, the patient was started on aspirin and given the degree of thrombocytosis; cytoreduction therapy with hydroxyurea was initiated. The patients' symptoms and platelet count improved over the next few days with hydroxyurea continued.

Despite previous studies revealing no evidence, this case demonstrates that *CALR* mutations can exist in patients presenting with stroke and a reasonable suspicion of ET; a situation similar to the very low incidence of *CALR* mutations in patients presenting with splanchnic vein thrombosis. 9 *CALR* mutational analysis should therefore be considered in the molecular diagnostic workup of stroke after exclusion of the *JAK2* V617F, particularly in the presence of a thrombocytosis, as a confirmatory finding will impact on future management for the prevention of recurring stroke and other thrombotic episodes.
